# Transcriptome and Physiological Analysis Reveals the Mechanism of Abscisic Acid in Regulating Cadmium Uptake and Accumulation in the Hyperaccumulator *Phytolacca acinosa* Roxb.

**DOI:** 10.3390/plants14101405

**Published:** 2025-05-08

**Authors:** Qin Xie, Wenting Xu, Qing Wang, Feihong Yao, Yachao Jiang, Haijia Cao, Wanhuang Lin

**Affiliations:** 1 College of Pharmacy, Xiangnan University, Chenzhou 423099, China; 2 Hunan Provincial Key Laboratory of Phytohormones and Growth Development, Hunan Agricultural University, Changsha 410128, China; 3 Biomedical Microbiome Research Laboratory, Xiangnan University, Chenzhou 423099, China

**Keywords:** abscisic acid, cadmium uptake and accumulation, *Phytolacca acinosa* Roxb., regulatory mechanism

## Abstract

Cadmium (Cd) is an extremely toxic heavy metal that can move from the soil to plants and enter the human body via the food chain, causing severe health issues for humans. Phytoremediation uses hyperaccumulators to extract heavy metals from polluted soil. Phytohormones, wildly used plant growth regulators, have been explored to improve phytoremediation efficiency. Abscisic acid (ABA) is also an essential regulator of plant tolerance to biotic and abiotic stresses, including heavy metal-induced toxicity. Previous research has revealed that *Phytolacca acinosa* Roxb. (*P. acinosa*) has a strong ability to enrich Cd and can be used as a Cd hyperaccumulator. In this study, physiological and biochemical analysis revealed that under Cd stress, exogenous ABA application alleviated oxidative stress, increased the Cd^2+^ concentration in *P. acinosa*, especially in the roots, and changed the phytohormone concentration in *P. acinosa*. Transcriptome analysis was conducted to explore the molecular mechanisms by which ABA regulates Cd uptake and accumulation in *P. acinosa*, and to further understand the regulatory role of ABA. The results show that ABA treatment affected gene expression in *P. acinosa* roots under Cd stress. This study identified 5788 differentially expressed genes (DEGs) (2541 up-regulated and 3247 down-regulated). Moreover, 96 metal transport-related DEGs, 54 phytohormone-related DEGs, 89 cell wall-related DEGs, 113 metal chelation-related DEGs, and 102 defense system-related DEGs cooperated more closely under exogenous ABA application to regulate Cd uptake and accumulation in *P. acinosa* under Cd stress. These results may help to elucidate the mechanisms by which ABA regulates Cd uptake and accumulation in plants, and provide a reference for developing a phytohormone-based strengthening strategy to improve the phytoremediation ability of other hyperaccumulators or accumulator species. The key genes involved in ABA’s regulation of Cd uptake and accumulation in *P. acinosa* need to be further analyzed and functionally verified. This may expand our understanding of the molecular regulatory mechanisms underlying heavy metal uptake and accumulation in hyperaccumulators.

## 1. Introduction

Soil is a crucial resource for human survival, and heavy metal pollution in soil has rapidly risen in recent decades, because of the rapid development of modern industrialization and agriculture [1]. Cd is an extremely toxic heavy metal that can move from the soil to plants and enter the human body via the food chain, causing severe health issues for humans [2].

Phytoremediation uses hyperaccumulators to extract heavy metals from polluted soils by harvesting plant parts [3,4]. This technique is widely regarded as an environmentally friendly and low-cost technique for the remediation of soil pollution caused by heavy metals, and has gained significant attention [5]. Recently, strategies have been used to improve the efficiency of phytoremediation, such as the application of plant growth regulators and bacteria, and the development of transgenic plants [6,7]. Phytohormones, widely used plant growth regulators, have been explored as a tool to improve phytoremediation efficiency. Phytohormones can promote plant tolerance to heavy metals and increase heavy metal uptake and accumulation, thereby improving the efficiency of phytoremediation [8,9].

As an important signaling molecule in plants, abscisic acid (ABA) not only regulates plant growth and development, but is also an essential regulator of plant tolerance to biotic and abiotic stresses, including heavy metal-induced toxicity [10,11]. ABA inhibits Cd transport into cells and increases antioxidant activity to alleviate Cd stress [12]. ABA can decrease Cd transport and alleviate Cd stress by inhibiting Cd loading in the xylem [13]. ABA reduces Cd toxicity by decreasing the Cd content in plant shoots, activating antioxidant systems, and reducing reactive oxygen species (ROS) [14]. These findings show that ABA inhibits the transport of heavy metals to shoots. However, many studies have proven that ABA can promote heavy metal accumulation. In *Solanum photeinocarpum*, ABA increases plant biomass and Cd content in the shoots [15]. In *Populus* × *canescens*, ABA can enhance the transport of plumbum (Pb) from roots to shoots [16,17]. A recent study on tall fescue found that combining ABA with salicylic acid (SA) significantly affected the transport of Cd from roots to shoots, leading to Cd accumulation in senescent leaves [18]. ABA has also been shown to increase the Cd content in *Solanum photeinocarpum*, thereby improving its phytoremediation ability [15]. These findings suggest that ABA regulates heavy metal uptake and accumulation, but this process is complex and varies among different species and heavy metals. If ABA is applied to accumulator species or hyperaccumulators, it may improve their resistance to heavy metal stress and increase their ability to uptake heavy metals. However, few studies have focused on this issue.

*P. acinosa* has demonstrated remarkable potential for the accumulation of heavy metals, particularly Cd and manganese (Mn) [19]. Our previous research revealed that *P. acinosa* has a strong ability to enrich Cd, and genes related to transcription factors, secondary metabolism, membrane proteins, ion transporters, and the defense system are involved in the response to Cd stress in roots [20]. Therefore, the present study aims to improve the phytoremediation capability of *P. acinosa*, and we address the question of whether ABA plays a role in regulating Cd uptake and accumulation in *P. acinosa*. In this study, *P. acinosa* seedlings were used as materials, and Cd stress was applied, in combination with exogenous ABA application, to investigate the effect of exogenous ABA on the physiological and molecular responses of *P. acinosa* seedlings to Cd stress, and to examine whether exogenous ABA application regulates Cd uptake and accumulation in *P. acinosa.* Physiological and biochemical analyses show that under Cd stress, exogenous ABA application could alleviate oxidative stress, increase the Cd^2+^ concentration in *P. acinosa*, especially in the roots, and change the phytohormone concentration in *P. acinosa*. Transcriptome analysis was conducted to understand the regulatory role of ABA and to explore the molecular mechanisms of ABA in regulating Cd uptake and accumulation in *P. acinosa*. As a result, under exogenous ABA application, genes associated with metal transport, phytohormones, the cell wall, metal chelation, and the defense system were differentially expressed; these genes cooperated more closely under exogenous ABA application to regulate Cd uptake and accumulation in *P. acinosa* under Cd stress. These findings will provide a reference for developing a phytohormone-based approach to improve the phytoremediation ability of other hyperaccumulators or accumulator species.

## 2. Results

### 2.1. Effects of ABA Treatment on Cd Uptake and Accumulation in P. acinosa Under Cd Stress

**Figure 1:**
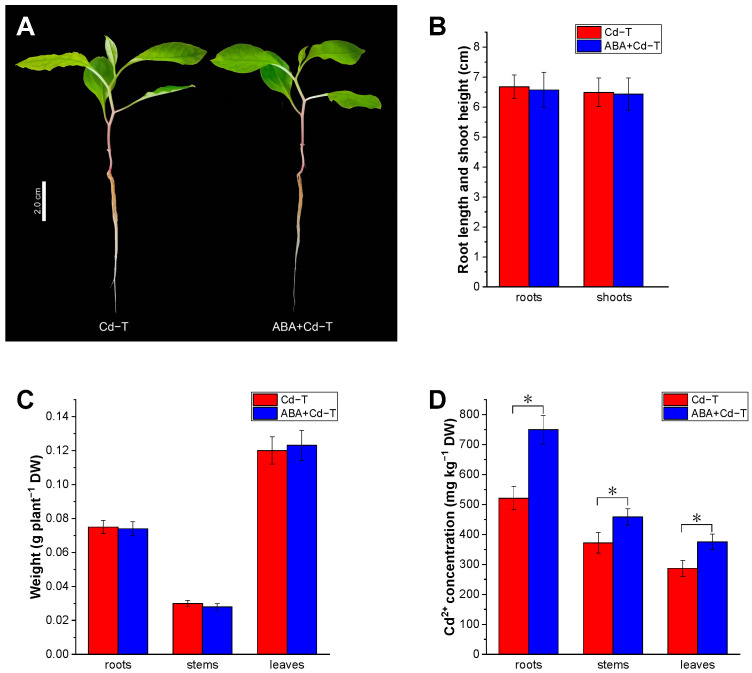
Effects of ABA treatment on Cd uptake and accumulation in *P*. *acinosa* under Cd stress. (**A**) Phenotype of seedlings; (**B**) root length and shoot height; (**C**) weight; (**D**) Cd^2+^ concentration. Data are shown as means ± SD of three biological replicates. * represents *p* < 0.05.

During ABA+Cd and Cd treatment, the root system developed well, and there was no significant difference between ABA+Cd treatment (ABA+Cd−T) samples and Cd treatment (Cd−T) samples. After 10 days of treatment with Cd and ABA+Cd, seedling growth and development also exhibited no significant differences between ABA+Cd−T and Cd−T samples (Figure 1A). The root length, shoot height, and dry weight of ABA+Cd−T and Cd−T samples were analyzed. The root lengths of ABA+Cd−T and Cd−T samples were 6.57 and 6.68 cm, respectively, and the shoot heights of ABA+Cd−T and Cd−T samples were 6.43 and 6.49 cm, respectively. The root lengths and shoot heights of the ABA+Cd−T samples did not differ significantly from those of the Cd−T samples (Figure 1B). The dry weights of the ABA+Cd−T samples were also comparable to those of the Cd−T samples (Figure 1C). The Cd^2+^ concentration of the ABA+Cd−T and Cd−T samples was also measured. The Cd^2+^ concentrations in the roots, stems, and leaves of ABA+Cd−T samples were 749.24, 458.60, and 375.26 mg kg^−1^. These values were higher than those found in the Cd−T samples, which were 521.11, 372.04, and 287.18 mg kg^−1^ for roots, stems, and leaves, respectively. Compared to the Cd−T samples, ABA increased the Cd^2+^ concentration in the roots, stems, and leaves by 43.78%, 23.27%, and 30.67%, respectively (Figure 1D).

### 2.2. Effects of ABA Treatment on Oxidative Stress of P. acinosa Under Cd Stress

**Figure 2:**
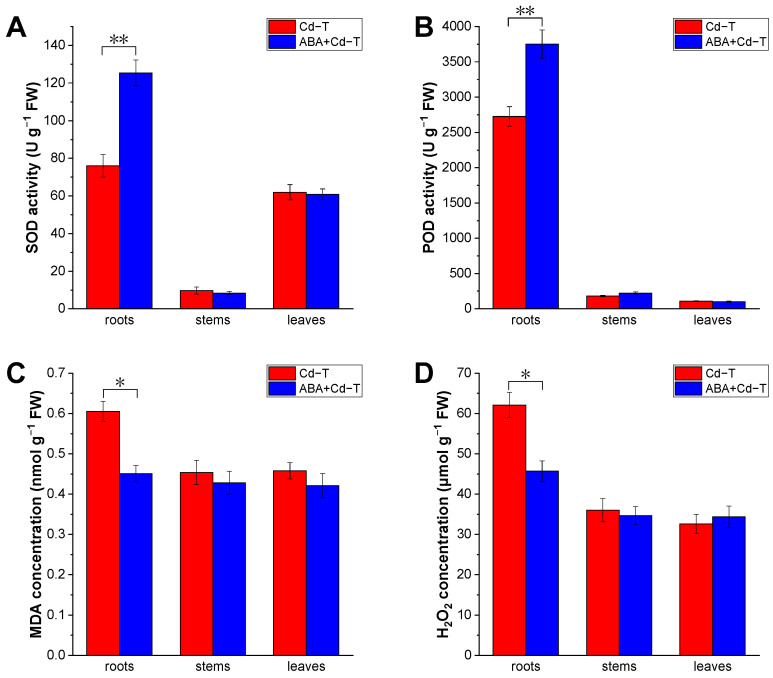
Effects of ABA treatment on oxidative stress of *P*. *acinosa* under Cd stress. (**A**) SOD activity; (**B**) POD activity; (**C**) MDA concentration; (**D**) H_2_O_2_ concentration. Data are shown as means ± SD of three biological replicates. * represents *p* < 0.05, ** represents *p* < 0.01.

To analyze the effects of ABA treatment on oxidative stress in *P. acinosa* seedlings under Cd stress, the superoxide dismutase (SOD) and peroxidase (POD) activities, as well as the malondialdehyde (MDA) concentration and hydrogen peroxide (H_2_O_2_) concentration, were detected in the ABA+Cd−T and Cd−T samples. The SOD activities were 125.35 and 76.04 U g^−1^ in the roots of the ABA+Cd−T and Cd−T samples, respectively (Figure 2A). Compared to the Cd−T samples, ABA significantly enhanced the SOD activity by 64.85% in the roots. However, the SOD activity in stems and leaves displayed minor differences between the Cd−T and ABA+Cd−T samples. The POD activities were 3751.99 and 2725.72 U g^−1^ in the roots of the ABA+Cd−T and Cd−T samples, respectively (Figure 2B). Compared to the Cd−T samples, ABA significantly increased the POD activity by 37.65% in the roots. The POD activity in the stems and leaves of the ABA+Cd−T samples was also comparable to that in the Cd−T samples. Additionally, the MDA concentration and H_2_O_2_ concentration were 0.61 nmol g^−1^ (Figure 2C) and 62.12 μmol g^−1^ (Figure 2D) in the roots of the Cd−T samples, and in the ABA+Cd−T samples, the MDA concentration and H_2_O_2_ concentration were 0.45 nmol g^−1^ (Figure 2C) and 45.69 μmol g^−1^ (Figure 2D) in the roots, respectively. Compared to the Cd−T samples, ABA significantly reduced the MDA concentration and H_2_O_2_ concentration in the roots by 26.45% and 26.22%, respectively, but did not decrease the concentrations of these components in the stems and leaves.

### 2.3. Effects of ABA Treatment on Phytohormone Concentration of P. acinosa Under Cd Stress

**Figure 3:**
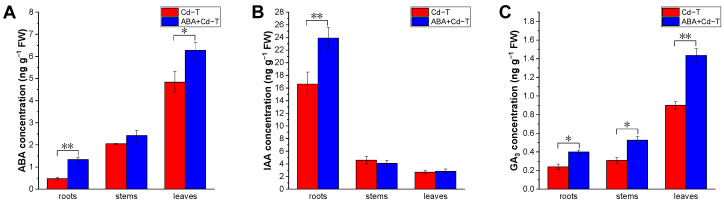
Effects of ABA treatment on phytohormone concentrations of *P. acinosa* under Cd stress. (**A**) ABA concentration; (**B**) IAA concentration; (**C**) GA_3_ concentration. Data are shown as means ± SD of three biological replicates. * represents *p* < 0.05, ** represents *p* < 0.01.

Since ABA may stimulate plant resistance to heavy metal stress, the ABA concentration was measured in the ABA+Cd−T and Cd−T samples. Whether exogenous ABA was applied or not, the trend of ABA concentration in *P. acinosa* seedlings was as follows: roots < stems < leaves. In the ABA+Cd−T samples, the ABA concentrations were 1.33, 2.43, and 6.28 ng g^−1^ in the roots, stems, and leaves, respectively, which were higher than those in the Cd−T samples, of 0.48, 2.05, and 4.84 ng g^−1^, respectively (Figure 3A).

In addition to ABA, the concentrations of IAA and GA_3_, which are known to regulate plant growth and development and respond to external stresses, were also measured in the ABA+Cd−T and Cd−T samples. In the ABA+Cd−T and Cd−T samples, the IAA concentration in *P. acinosa* seedlings was as follows: roots > stems > leaves. The IAA concentrations were 23.92, 4.08, and 2.83 ng g^−1^ in the roots, stems, and leaves of the ABA+Cd−T samples, and 16.63, 4.58, and 2.68 ng g^−1^ in the roots, stems, and leaves of the Cd−T samples, respectively. The results revealed that the IAA concentration in the roots of the ABA+Cd−T samples was 1.44-fold that in the Cd−T samples, whereas it did not change significantly in the stems and leaves (Figure 3B). The trend of GA_3_ concentration in *P. acinosa* seedlings was consistent with that of ABA concentration, as follows: roots < stems < leaves. The GA_3_ concentrations were 0.40, 0.53, and 1.44 ng g^−1^ in the roots, stems, and leaves of the ABA+Cd−T samples, respectively, which were 1.67-, 1.70-, and 1.60-fold higher than those in the Cd−T samples, respectively (Figure 3C).

The results demonstrated that exogenous ABA application increased the endogenous ABA and GA_3_ concentrations in the roots, stems, and leaves, as well as the endogenous IAA concentration in the roots. Additionally, in the ABA+Cd−T samples, the ratio of ABA/IAA in the roots was 0.056 (1.33/23.92), which was higher than that of 0.029 (0.48/16.63) in the Cd−T samples. In addition, the ratio of ABA/GA_3_ in the roots of the ABA+Cd−T samples was 3.33 (1.33/0.40), which was higher than that of 2.0 (0.48/0.24) in the Cd−T samples.

### 2.4. De Novo Transcriptome Sequencing and Analysis of DEGs Between ABA+Cd−T and Cd−T Samples in P. acinosa Roots

The roots of the ABA+Cd−T and Cd−T samples were collected for RNA extraction. De novo sequencing was performed using Illumina HiSeq 4000, and the ABA+Cd−T and Cd−T samples were evaluated in three biological replicates. The Cd−T samples yielded 37,184,932, 42,969,078, and 41,966,768 clean reads, with over 93% Q30 bases (Table 1). The ABA+Cd−T samples yielded 37,209,394, 38,817,700, and 46,448,560 clean reads, with over 93% Q30 bases (Table 1).

**Figure 4:**
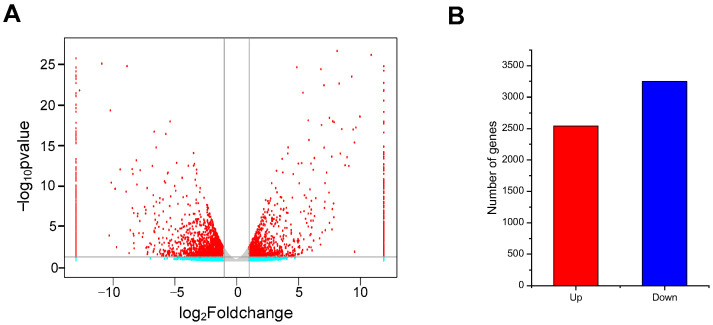
(**A**) Volcano analysis of DEGs. Red dots represent significant change; green dots represent non-significant change; gray dots represent no significant change. (**B**) Statistics of up- and down-regulated DEGs. Red column represents up-regulated and blue column represents down-regulated.

The fragments per kilobase per million (FPKM) was used to estimate gene expression, and DEGs were identified in the Cd−T and ABA+Cd−T samples based on the threshold criteria of |Log_2_FoldChange| ≥ 1 and *p*-value ≤ 0.05. Using these criteria, 5788 DEGs (2541 up-regulated and 3247 down-regulated) were identified (Figure 4A,B).

### 2.5. Metal Transport-Related Gene Expression Differences Between ABA+Cd−T and Cd−T Samples in P. acinosa Roots

**Figure 5:**
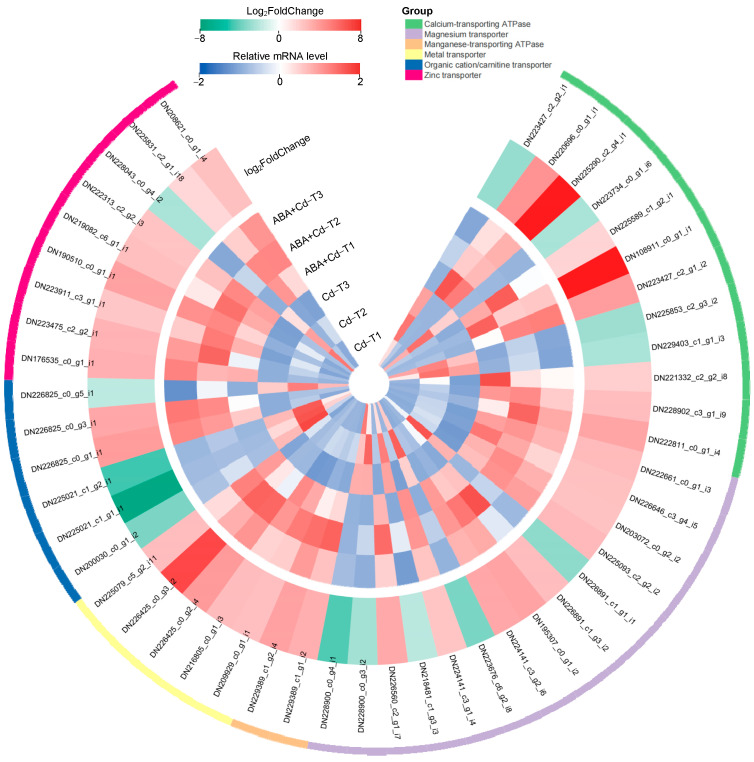
Expression profiles of metal transport-related DEGs (|log_2_FoldChange| ≥ 1 and *p*-value ≤ 0.05).

Metal transport is an important biological process for Cd accumulation in plants [21]. This study observed a higher Cd^2+^ concentration in the roots, stems, and leaves of ABA+Cd−T samples than in Cd−T samples. Transcriptome analysis found 96 common metal transport-related DEGs (73 up-regulated and 23 down-regulated) between the ABA+Cd−T and Cd−T samples. Their expression profiles are shown in Figure 5 and Table 2. Among the 96 DEGs, there were 12 calcium-transporting ATPase DEGs (eight up-regulated and four down-regulated), 14 magnesium (Mg) transporter DEGs (nine up-regulated and five down-regulated), two Mn-transporting ATPase up-regulated DEGs, five metal transporter up-regulated DEGs, six organic cation/carnitine transporter DEGs (two up-regulated and four down-regulated), nine zinc transporter DEGs (eight up-regulated and one down-regulated), and 48 ABC transporter DEGs (39 up-regulated and nine down-regulated).

The *Nramp* family has been reported as transporters of Mn, Ferrum (Fe), and Cd, playing a crucial role in Cd uptake and transport [22]. In this study, the expression of five *Nramp* family members was up-regulated in the ABA+Cd−T samples, including *Nramp2* (DN209929_c0_g1_i1), *Nramp3* (DN216805_c0_g1_i3), *Nramp4* (DN226425_c0_g2_i4), *Nramp5* (DN226425_c0_g3_i2), and *Nramp6* (DN225079_c5_g2_i11), with *Nramp5* exhibiting the most significant up-regulation (Log_2_FoldChange = 6.39). Moreover, eight *ZIP* family members showed high expression levels; *ZIP4* (DN190510_c0_g1_i1), *ZIP1* (DN176535_c0_g1_i1), *ZIP2* (DN223475_c2_g2_i1) exhibited the most significant up-regulation, by 3.31-, 2.80-, and 2.70-fold changes (Log_2_FoldChange level), respectively.

This study discovered 48 ABC transporter DEGs, including three *ABCA* subfamily up-regulated DEGs, 11 *ABCB* subfamily DEGs (eight up-regulated and three down-regulated), 10 *ABCC* subfamily DEGs (eight up-regulated and two down-regulated), one *ABCD* subfamily up-regulated DEG, one *ABCE* subfamily up-regulated DEG, four *ABCF* subfamily up-regulated DEGs, 13 *ABCG* subfamily DEGs (10 up-regulated and three DEGs down-regulated), and five *ABCI* subfamily DEGs (four up-regulated and one down-regulated) (Table 2). *ABCB8* (DN220815_c0_g1_i1), *ABCB17* (DN189123_c0_g1_i1), *ABCB19* (DN173660_c0_g1_i1), and *ABCG42* (DN228476_c1_g5_i1) exhibited the most significant up-regulation, by 4.31-, 4.94-, 4.42-, and 4.41-fold changes (Log_2_FoldChange level), respectively. In the ABA+Cd−T samples, ABC transporter DEGs might have directly participated in the Cd uptake and transport, and might also have been involved in mediating the transport of ABA in the plants, which is worthy of further in-depth study.

### 2.6. Phytohormone-Related Gene Expression Differences Between ABA+Cd−T and Cd−T Samples in P. acinosa Roots

**Figure 6:**
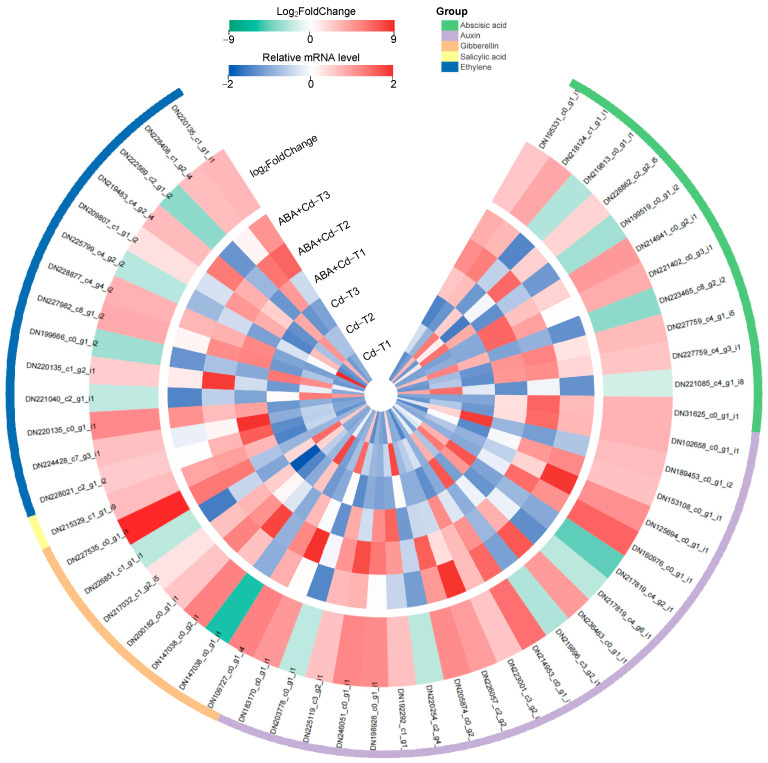
Expression profiles of phytohormone-related DEGs (|log_2_FoldChange| ≥ 1 and *p*-value ≤ 0.05).

To investigate the regulatory role of phytohormones in Cd uptake and accumulation in *P. acinosa* under exogenous ABA application, the expression patterns of DEGs associated with phytohormone biosynthesis, metabolism, transport, and signaling pathways were analyzed. This study identified 54 phytohormone-related DEGs, with 39 up-regulated and 15 down-regulated, and their expression profiles are shown in Figure 6. A total of 12 ABA-related DEGs were found, including four ABA-insensitive DEGs (three up-regulated and one down-regulated) and six ABA receptor DEGs (four up-regulated and two down-regulated). The *PYL* family is a core regulator of ABA signaling, and has been widely studied in *Arabidopsis* and rice [23]. In this study, *PYL3* (DN227759_c4_g1_i5), *PYL4* (DN221402_c0_g3_i1), *PYL5* (DN214941_c0_g2_i1), and *PYL8* (DN227759_c4_g3_i1) were up-regulated by 2.73-, 3.36-, 4.03-, and 2.34-fold changes (Log_2_FoldChange level), respectively, whereas *PYL9* (DN223465_c8_g2_i2) was down-regulated by a 3.46-fold change (Log_2_FoldChange level). In addition, one ABA 8′-hydroxylase down-regulated DEG (DN221085_c4_g1_i8), and one abscisic-aldehyde oxidase up-regulated DEG (DN31625_c0_g1_i1) were discovered, which may have led to the increased ABA level in *P. acinosa* roots.

Meanwhile, this study discovered 20 auxin-related DEGs, including two auxin homeostasis up-regulated DEGs, three auxin efflux carrier up-regulated DEGs, and three auxin transporter protein DEGs (one up-regulated and two down-regulated); these DEGs may be important for maintaining the auxin concentration in *P. acinosa* roots. Two auxin-binding protein DEGs (one up-regulated and one down-regulated), seven auxin-responsive protein DEGs (six up-regulated and one down-regulated), and three auxin response factor DEGs (two up-regulated and one down-regulated) were identified. Further analysis found that three *Auxin/IAA* family members, *IAA1* (DN223001_c3_g2_i3), *IAA21* (DN226057_c2_g2_i1), and *IAA27* (DN205874_c0_g2_i1), were up-regulated by 3.30-, 3.89-, and 4.58-fold changes (Log_2_FoldChange level), respectively. Three *SAUR* family members, *SAUR32* (DN192292_c1_g1_i1), *SAUR36* (DN198928_c0_g1_i1), and *SAUR71* (DN246051_c0_g1_i1), were up-regulated by 2.51-, 4.54-, and 4.78-fold changes (Log_2_FoldChange level), respectively; these DEGs contribute to auxin signal transduction. Seven GA-related DEGs were identified, including two GA beta-dioxygenase up-regulated DEGs, two GA oxidase proteins DEGs (one up-regulated and one down-regulated), two GA receptor DEGs (one up-regulated and one down-regulated), and one GA-regulated protein up-regulated DEG. This study also identified 14 ethylene-related DEGs, including one ethylene-response factor up-regulated DEG and 13 ethylene-responsive transcription factor DEGs (nine up-regulated and four down-regulated). The *ERF* family is a major ethylene-responsive factor, and further analysis found that four *ERF* family members, *ERF012* (DN227982_c8_g1_i2), *ERF062* (DN228877_c4_g4_i2), *ERF114* (DN209807_c1_g1_i2), and *ERF115* (DN219483_c4_g2_i4) were up-regulated by 3.41-, 3.11-, 1.22-, and 2.69-fold changes (Log_2_FoldChange level), respectively. In addition, one SA-binding protein DEG was up-regulated in the ABA+Cd−T samples.

### 2.7. Cell Wall-Related Gene Expression Differences Between ABA+Cd−T and Cd−T Samples in P. acinosa Roots

**Figure 7:**
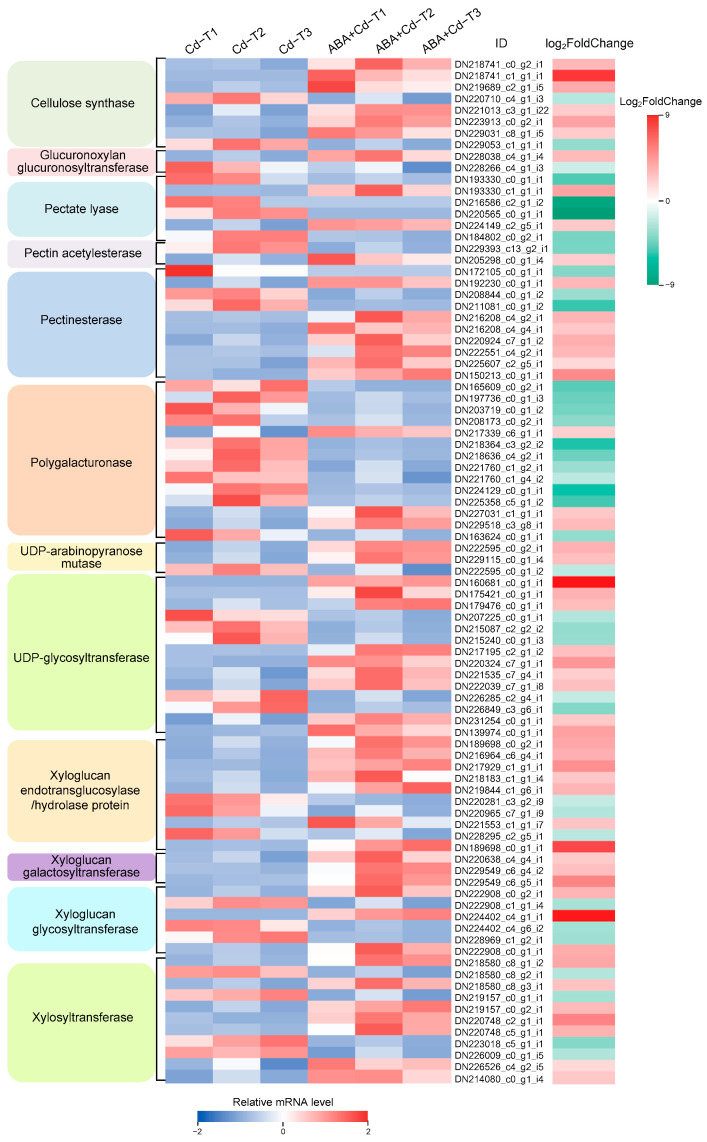
Expression profiles of cell wall-related DEGs (|log_2_FoldChange| ≥ 1 and *p*-value ≤ 0.05).

The cell wall can adsorb and intercept heavy metals, playing a dual role in heavy metal accumulation in hyperaccumulators [24]. In this study, 89 cell wall-related DEGs (51 up-regulated and 38 down-regulated) were identified, and their expression profiles are shown in Figure 7.

Eight cellulose synthase DEGs (six up-regulated and two down-regulated) were found; *CSLD2* (DN218741_c0_g2_i1), *CSLD3* (DN229031_c8_g1_i5), *CSLE1* (DN219689_c2_g1_i5), *CSLE6* (DN221013_c3_g1_i22), *CESA3* (DN223913_c0_g2_i1), and *CESA8* (DN218741_c1_g1_i1) were up-regulated by 2.79-, 2.09-, 3.34-, 2.11-, 3.58-, and 7.42-fold changes (Log_2_FoldChange level), respectively. These DEGs are key enzymes that catalyze cellulose synthesis and form the cell wall skeleton. Ten xyloglucan endotransglucosylase/hydrolase protein DEGs (seven up-regulated and three down-regulated) were identified. *XTH1* (DN217929_c1_g1_i1), *XTH5* (DN219844_c1_g6_i1), *XTH8* (DN189698_c0_g1_i1), *XTH9* (DN189698_c0_g2_i1), *XTH22* (DN216964_c6_g4_i1), *XTH823* (DN218183_c1_g1_i4), and *XTH33* (DN221553_c1_g1_i7) were up-regulated by 4.31-, 2.92-, 6.80-, 3.42-, 3.11-, 2.24-, and 2.30-fold changes (Log_2_FoldChange level), respectively; these DEGs are involved in cell wall modifications.

In addition, three xyloglucan galactosyltransferase DEGs, three xyloglucan glycosyltransferase DEGs, seven xylosyltransferase DEGs, and three polygalacturonase DEGs were up-regulated, and these DEGs are involved in cell wall construction and modification. Four pectate lyase DEGs and one pectin acetylesterase DEG were down-regulated; these contribute to maintaining the integrity of the cell wall skeleton. Meanwhile, seven pectinesterase DEGs were up-regulated, suggesting that the increased activity of pectin esterase promotes the conversion of pectin to pectinic acid, allowing more Cd to be bound to the cell wall. Additionally, two glucuronoxylan glucuronosyltransferase DEGs (one up-regulated and one down-regulated), three UDP-arabinopyranose mutase DEGs (two up-regulated and one down-regulated), and 14 UDP-glycosyltransferase DEGs (nine up-regulated and five down-regulated) were found. These results indicate that greater expression of several DEGs involved in cell wall synthesis, organization, and modification was induced by exogenous ABA application.

### 2.8. Metal Chelation-Related Gene Expression Differences Between ABA+Cd−T and Cd−T Samples in P. acinosa Roots

**Figure 8:**
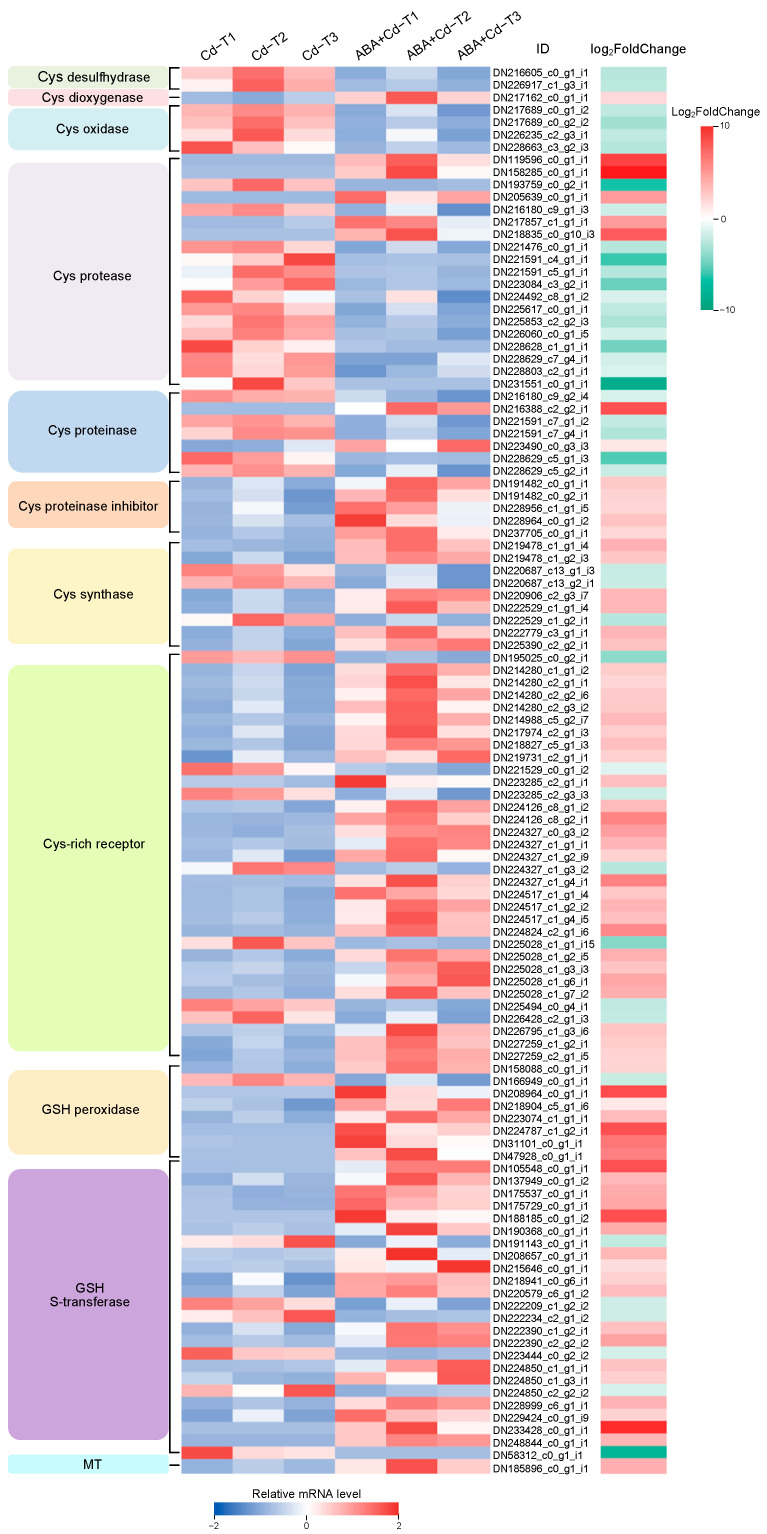
Expression profiles of metal chelation-related DEGs (|log_2_FoldChange| ≥ 1 and *p*-value ≤ 0.05).

This study identified 113 metal chelation-related DEGs (71 up-regulated and 42 down-regulated) among the ABA+Cd−T and Cd−T samples of *P. acinosa* roots, and their expression profiles are shown in Figure 8.

In the cysteine (Cys) synthesis and metabolism pathway, two Cys desulfhydrase down-regulated DEGs, one Cys dioxygenase up-regulated DEG, four Cys oxidase down-regulated DEGs, and nine Cys synthase DEGs (six up-regulated and three down-regulated) were found, suggesting that exogenous ABA application increased the chelation of Cys and Cd by modulating Cys synthesis and metabolism in the *P. acinosa* roots. Meanwhile, 19 Cys protease DEGs (five up-regulated and 14 down-regulated), seven Cys proteinase DEGs (two up-regulated and five down-regulated), five Cys proteinase inhibitor up-regulated DEGs, and 33 Cys-rich receptor DEGs (26 up-regulated and seven down-regulated) were found. These findings indicate that the synergistic effect of Cys protease, Cys proteinase, and Cys protease inhibitor regulated the response of *P. acinosa* to Cd stress under exogenous ABA application. In addition, eight glutathione (GSH) peroxidase DEGs (seven up-regulated and one down-regulated) and 24 GSH S-transferase DEGs (18 up-regulated and six down-regulated) were identified, indicating that under exogenous ABA application, GSH peroxidase and GSH S-transferase may also be important for *P. acinosa* responses to Cd stress.

Among these metal chelation-related DEGs, DN158285_c0_g1_i1 (Cys protease), DN233428_c0_g1_i1 (GSH S-transferase), and DN119596_c0_g1_i1 (Cys protease) were significantly up-regulated, by 9.78-, 9.06-, and 8.20-fold changes (Log_2_FoldChange level), respectively. Moreover, DN231551_c0_g1_i1 (Cys protease), DN58312_c0_g1_i1 (GSH S-transferase), and DN193759_c0_g2_i1 (Cys protease) were significantly down-regulated by 8.60-, 7.79-, and 6.34-fold changes (Log_2_FoldChange level), respectively.

### 2.9. Defense System-Related Gene Expression Differences Between ABA+Cd−T and Cd−T Samples in P. acinosa Roots

Based on previous studies, it is possible that the expression of defense system-related genes could be changed to improve the tolerance of plants to heavy metal stress [25]. ABA participates in regulating the defense system and alleviating heavy metal-induced stress when plants are exposed to heavy metals [26].

**Figure 9:**
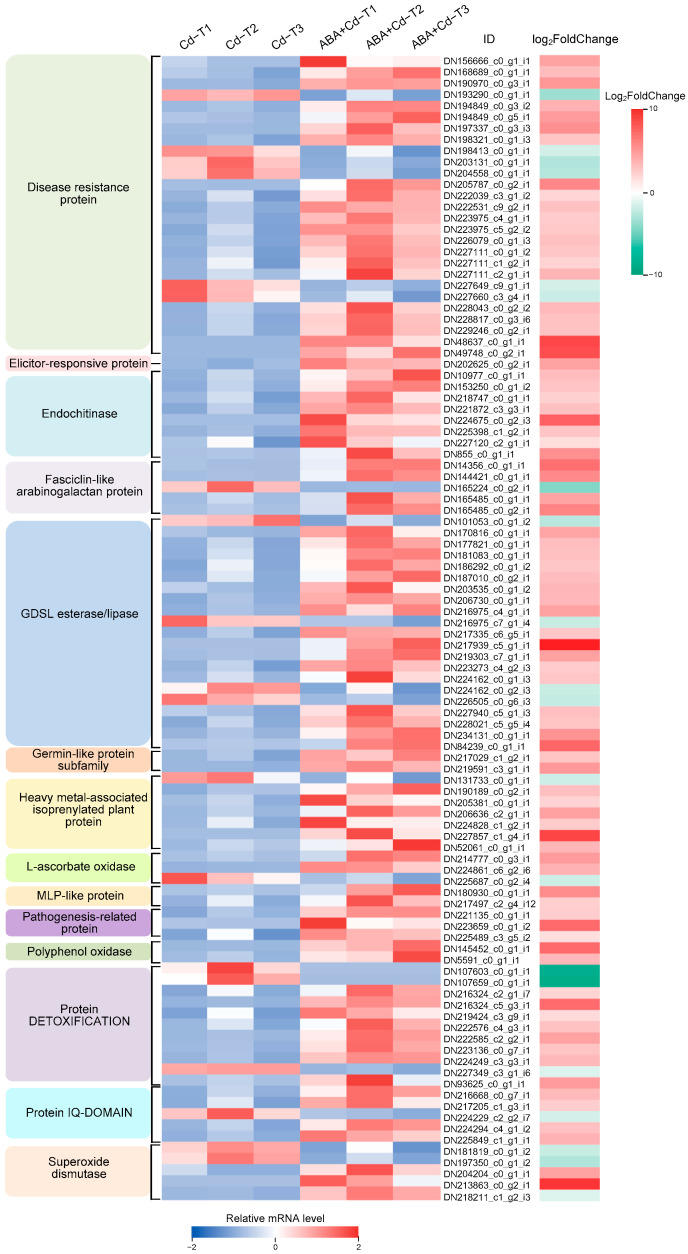
Expression profiles of defense system-related DEGs (|log_2_FoldChange| ≥ 1 and *p*-value ≤ 0.05).

In this study, 102 defense system-related DEGs (83 up-regulated and 19 down-regulated) were identified (Figure 9). Disease resistance proteins were associated with the greatest number of DEGs (21 up-regulated and six down-regulated). GDSL esterase/lipase (17 up-regulated and four down-regulated) were associated with the second greatest number of DEGs. Protein DETOXIFICATION (eight up-regulated and three down-regulated) was associated with the third greatest number of DEGs. Meanwhile, endochitinase was associated with eight up-regulated DEGs, and heavy metal-associated isoprenylated plant proteins were associated with seven DEGs (six up-regulated and one down-regulated). Five protein IQ-DOMAIN DEGs (four up-regulated and one down-regulated), five superoxide dismutase DEGs (two up-regulated and three down-regulated), and five fasciclin-like arabinogalactan protein DEGs (four up-regulated and one down-regulated) were found. Additionally, three L-ascorbate oxidase DEGs, three pathogenesis-related protein DEGs, two MLP-like protein DEGs, two polyphenol oxidase DEGs, two germin-like protein subfamily DEGs, and one elicitor-responsive protein DEG were identified. The results show that most up-regulated DEGs were involved in plant defense mechanisms. Among these DEGs, DN217939_c5_g1_i1 (GDSL esterase/lipase), DN213863_c0_g2_i1 (superoxide dismutase), and DN227857_c1_g4_i1 (heavy metal-associated isoprenylated plant protein) were significantly up-regulated, by 9.56-, 8.69-, and 8.07-fold changes (Log_2_FoldChange level), respectively. Moreover, a small number of defense system-related DEGs were down-regulated: DN107659_c0_g1_i1 (protein DETOXIFICATION), DN107603_c0_g1_i1 (protein DETOXIFICATION), and DN165224_c0_g2_i1 (fasciclin-like arabinogalactan protein) were significantly down-regulated, by 8.65-, 8.25-, and 4.13-fold changes (Log_2_FoldChange level), respectively.

### 2.10. Real Time-PCR Validation of DEGs

**Figure 10:**
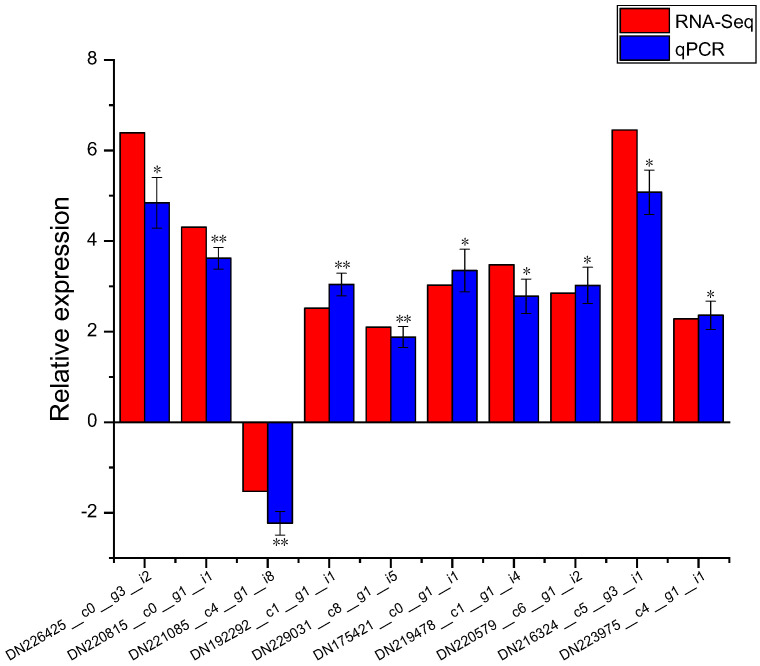
Expression patterns of 10 DEGs confirmed by real-time PCR (log_2_Foldchange levels). Data are shown as means ± SD of three biological replicates. Based on Dunnett’s test, * represents *p* < 0.05, ** represents *p* < 0.01.

Ten DEGs were selected to determine their gene expression by real time-PCR, including two metal transport-related DEGs (DN226425_c0_g3_i2 and DN220815_c0_g1_i1), two phytohormone-related DEGs (DN221085_c4_g1_i8 and DN192292_c1_g1_i1), two cell wall-related DEGs (DN229031_c8_g1_i5 and DN175421_c0_g1_i1), two metal chelation-related DEGs (DN219478_c1_g1_i4 and DN220579_c6_g1_i2), and two defense system-related DEGs (DN216324_c5_g3_i1 and DN223975_c4_g1_i1). The results demonstrate that under Cd stress, the expression levels of these 10 DEGs were significantly changed by exogenous ABA application, and the expression patterns are similar to the RNA-Seq data (Figure 10), suggesting that the RNA-Seq results are reliable.

## 3. Discussion

### 3.1. ABA Treatment Regulated Physiological and Biochemical Processes in P. acinosa Under Cd Stress

ABA is an essential phytohormone involved in regulating plant responses to various abiotic stresses, such as cold, drought, heavy metals, and salinity [27,28,29,30]. In this study, ABA+Cd−T and Cd−T samples were used to analyze the effects of ABA on Cd uptake and accumulation in *P. acinosa*, and the results revealed that ABA exhibited important functions in these processes. After ABA treatment, the root length, shoot height, and dry weight of *P. acinosa* seedlings did not change significantly under Cd stress. The results revealed that *P. acinosa* seedlings grew well, without differences between the ABA+Cd−T and Cd−T samples. The Cd^2+^ concentration results demonstrated that ABA increased the Cd^2+^ concentration in roots, stems, and leaves by 43.78%, 23.27%, and 30.67%, respectively. These results suggest that ABA can promote Cd uptake and accumulation in *P. acinosa*.

Abiotic stress can cause plants to produce ROS, which can trigger oxidative damage to cell membranes, proteins, lipids, and DNA [31]. In this study, the effect of oxidative stress indicated that the SOD and POD activities were higher in the roots of the ABA+Cd−T samples than in the roots of the Cd−T samples. These results revealed that ABA significantly induced SOD and POD activities in the roots, implying that ABA alleviated stress damage by increasing SOD and POD activities, which are crucial for maintaining redox homeostasis in roots.

Meanwhile, the MDA and H_2_O_2_ concentrations in the roots of the ABA+Cd−T samples were lower than in the roots of the Cd−T samples. The results showed that exogenous ABA application decreased MDA and H_2_O_2_ concentrations in the roots, indicating that ABA reduced the excessive accumulation of H_2_O_2_ to directly alleviate oxidative stress in the plants, increased antioxidant levels in the roots, and alleviated membrane lipid peroxidation. We speculate that since exogenous ABA application promoted Cd uptake and accumulation in *P. acinosa,* it could also maintain redox homeostasis and alleviate oxidative stress caused by Cd stress, reducing the toxicity of Cd to seedlings and improving their tolerance to Cd stress, and this is why, in the ABA+Cd−T samples, increased Cd accumulation did not affect the growth of *P. acinosa.*

In addition, transcriptome analysis found that exogenous ABA application induced a wide series of defense mechanisms in *P. acinosa* roots, which were also important mechanisms for enabling the plants to alleviate Cd stress, and in this process, complex interactions among endogenous phytohormones might have played an important role.

### 3.2. Phytohormone Signal Transduction Pathway Positively Responds to Cd Stress

Phytohormones, including ABA, auxin, cytokinin, GA, ethylene, SA, brassinosteroids (BRs), and jasmonates (JAs), are major regulators of plant growth, development, and stress responses [32,33]. Under exogenous phytohormone application, endogenous phytohormone concentration and signaling can regulate plant responses to heavy metal stress [21,34].

Some studies have reported that SA treatment can elevate the antioxidant enzyme activity of cauliflower and alleviate cell damage under Cd stress [35]; it can also improve photosynthetic capacity, enhance the antioxidant system, and regulate the uptake and distribution of Cd in plants [36,37]. Research on *Helianthus tuberosus* has revealed that SA can increase the Cd content and transportation factor, thereby improving its phytoremediation ability to Cd-contaminated soil [38]. GA_3_ enhanced the ability of the green algae *Chlorella Vulgaris* to adapt to low level of Pb and Cd [39]. Exogenous GA_3_ application improved root morphology and reduced both Cd uptake and root crown translocation, alleviating the toxic effects of Cd on lettuce leaves [40]. ABA, known as the “stress phytohormone”, is vital for plant responses to various biotic and abiotic stresses, including Cd stress [10]. Under Cd stress, ABA biosynthesis can be induced in several species, such as rice [41], potato [42], and *Sedum alfredii* [21], and ABA also plays an important role in various plant processes, including redox homeostasis and Cd transport [43].

In this study, exogenous ABA application enhanced endogenous ABA and GA_3_ concentrations in the roots, stems, and leaves of *P. acinosa*. It also increased the endogenous IAA concentration in the roots, but did not affect the endogenous IAA concentration in the leaves and stems. Accordingly, we speculated that when *P. acinosa* is under Cd stress, exogenous ABA application can rapidly generate ABA transmission signals and initiate a stress resistance mechanism following perceived stress. In addition, the ABA/IAA and ABA/GA_3_ ratios in the roots of the ABA+Cd−T samples were higher than that in the Cd−T samples. The results indicate that phytohormone concentrations and the ratios of different phytohormones can regulate downstream gene expression, acting as important players in the Cd uptake and accumulation in *P. acinosa* under Cd stress.

Meanwhile, transcriptome analysis found 54 phytohormone-related DEGs among the ABA+Cd−T and Cd−T samples, which were associated with ABA, IAA, GA, and ethylene, suggesting that the phytohormone signal transduction pathway positively responds to Cd stress.

### 3.3. Metal Transporter Proteins Play an Important Role in Cd Uptake and Accumulation in P. acinosa

In plants, heavy metal transporters are essential for heavy metal transport and accumulation [44]. Cd, a harmful non-essential element, has no corresponding metal transport channel in plants, and actively enters plant cells through uptake systems for other elements [45]. In plants, the *ZIP* family and *Nramp* family play important roles in Cd uptake and transport [46]. IRT1 (*ZIP* family member) was found to be involved in Cd uptake by the hyperaccumulator *Thlaspi caerulescens* [47]. ZIP4 has a transport function of Cd in yeast [48], and in our results, it was up-regulated in the ABA+Cd−T samples. OsNRAMP1 and OsNRAMP5 have been suggested to contribute to Cd uptake and transport in rice [22,49], and in this study, five *Nramp* family members were significantly up-regulated in the ABA+Cd−T samples; these findings imply that the *ZIP* family and *Nramp* family members could play important roles in ABA’s promotion of Cd uptake and transport in *P. acinosa*.

In plants, the ABC transporter family is one of the largest and most diverse protein groups, actively participating in the transport of metal between biological membranes [44]. Previous studies have suggested that ABC transporters are associated with the excretion of metals from cells and the transportation of metals to vacuoles in cells [50]. ABC transporters were found to respond to Zn signaling in the hyperaccumulator *Thlaspi caerulescens* [51]. The ABCB and ABCC subfamilies are usually localized in the tonoplast, which can transport Cd complexes from the cytoplasm to the vacuole for sequestration to improve the tolerance of plants to Cd [52,53]. Transcriptome studies found that the *ABCB* subfamily responded to Cd in *Sedum alfredii* Hance [54]. ABCC2 has been reported to be involved in intravesicular transport and sequestration of Cd in *L. chuanxiong*, as well as being a key factor in enhancing Cd tolerance and accumulation capacity [55]. Furthermore, in *Arabidopsis*, ABCC1 and ABCC2 have also been reported to participate in the sequestration of Cd [56]. In our results, under exogenous ABA application, eight *ABCB* subfamily DEGs and eight *ABCC* subfamily DEGs were up-regulated, and *ABCC1* and *ABCC2* were significantly up-regulated, by 3.45- and 3.12-fold changes (Log_2_FoldChange level), respectively, suggesting that they may play an important role in Cd transport and accumulation in *P. acinosa* under exogenous ABA application.

### 3.4. ABA Treatment May Enhance the Cell Wall Structure of the Root System

The cell wall can mediate the compartmentalization of heavy metals, intercept them outside the cell, and reduce their toxicity to cells [57]. On the other hand, it may participate in the apoplast transportation of heavy metals, effectively inhibiting the transportation of heavy metals to the symplast [58].

Plant cell walls are composed of several components, mainly cellulose, hemicellulose, and pectin [59], and the components of cell walls can affect their adsorption capacity. Plants’ capacity to absorb heavy metals can be improved by changing their cell wall components [60]. Several genes regulate the metabolism of these components to modulate cell walls under heavy metal stress. Xyloglucan is the most crucial hemicellulose polysaccharide, and xyloglucan synthase requires the participation of different glycosyltransferases. Xyloglucan endotransglucosylases/hydrolases are key enzymes involved in plant cell wall remodeling; they can remold cell walls by modifying the cellulose-xyloglucan complex structure of plant cell walls through the catalysis of xyloglucan molecule cleavage and reconnection [61]. Additionally, phytohormones play a role in cell wall modification, and the creation of cell walls is considered a fundamental event downstream of auxin regulation [62]. In our study, under exogenous ABA application, several DEGs associated with cell wall synthesis and composition were found, including cellulose synthase, xyloglucan endotransglucosylase/hydrolase protein, xyloglucan galactosyltransferase, and xyloglucan glycosyltransferase genes. These results revealed that ABA promoted cell wall synthesis in *P. acinosa* roots and changed the polysaccharide components in the cell wall. Combining these results with those of previous studies, it can be concluded that this change can promote the synthesis of metal chelates, especially Cys, and the binding of GSH to Cd, providing more energy that allows for greater Cd accumulation in *P. acinosa*.

The increased activity of pectinesterase can increase the level of de-esterified pectin, resulting in increased negative charges to bind Cd [63]. In our results, seven pectinesterase genes were up-regulated in the ABA+Cd−T samples, suggesting that these can allow more Cd to be bound to the cell wall. These findings indicate that ABA treatment influences enzyme activities and signaling pathways, forming complex interaction networks that dynamically coordinate cell wall synthesis, modification, and remodeling.

### 3.5. Phytohormone Signaling May Regulate Chelating Agents Involved in Cd Stress Response

In plants, heavy metal chelation and sequestration are important mechanisms involved in the uptake and accumulation of both essential and non-essential heavy metals [64]. Chelating agents can chelate heavy metals, thereby preventing their contact with organelles. GSH, phytochelatins (PCs), Cys, metallothionein (MT), nicotinamide (NA), and organic acids (OA) are the primary chelating agents in plants [55]. Some chelates can bind to metals and be transported by specific channels to mediate the transportation of metals, while other chelates can bind to metals, allowing metals to be compartmentalized extracellularly or in vacuoles, or allowing their valence state to be changed to reduce metal toxicity [44]. Studies on rice have demonstrated that GSH S-transferase is involved in mediating the binding of GSH to Cd, and increased GSH S-transferase gene expression contributes to more dynamic binding of Cd to GSH and its retention in roots, while alleviating the toxic effects of Cd on rice [65]. In this study, 18 GSH S-transferase DEGs and seven GSH peroxidase DEGs were up-regulated, suggesting that more Cd could bind to GSH and be retained in the *P. acinosa* roots under exogenous ABA application.

The biosynthesis and distribution of chelators requires a complex phytohormonal regulatory network [66]. Recent studies on the effects of phytohormones on metal chelation have mainly focused on PC compounds. Some studies have shown that phytohormones can participate in PC biosynthesis [64]. In the algae *Chlorella vulgaris*, BR increased the phytochelatin content under Pb stress [67]. In potato tubers, ABA was found to be involved in the regulation of PC biosynthesis [68]. The PC biosynthesis pathway is related directly to GSH production and the availability of Cys and other sulfur (S)-containing molecules [66]. It has also been found that ABA can increase the generation of GSH and PCs, along with the vacuolar fixation capacity of the conjugated complex [27]. This suggests that phytohormones play a critical role, both directly and indirectly, in the regulation of chelator biosynthesis.

In our study, under exogenous ABA application, several DEGs related to the synthesis and metabolism of Cys and GSH were found, suggesting that the high Cd levels observed in *P. acinosa* can be attributed to the increased efficiency of chelation mechanisms, and it can be speculated that chelating agents involved in Cd stress responses may be regulated by phytohormone signaling.

## 4. Materials and Methods

### 4.1. Plant Materials and Growth Conditions

*Phytolacca acinosa* Roxb. seeds were treated with 98% concentrated sulfuric acid to carbonize the seed shell for 10 min, and then washed 5–6 times with distilled water. Moist sand was placed in culture pots, and *P. acinosa* seeds were evenly sprinkled on the moist sand. A plastic wrap was used to maintain humidity and temperature. The seeds were incubated in a greenhouse with a 16 h light/8 h dark cycle at 22 °C. After five days, the seeds were germinated, and the seedlings were irrigated with ½ Hoagland solution.

### 4.2. Cd Treatments and ABA+Cd Treatments

*P. acinosa* seedlings (20 days old) with four true leaves were transplanted into an 800 mL hydroponic box (120 mm × 80 mm × 110 mm) with ½ Hoagland solution for hydroponic experiments. Two days later, Cd treatment (Cd−T, 10 mg L^−1^ Cd^2+^) and ABA+Cd treatment (ABA+Cd−T, 0.2 mg L^−1^ ABA and 10 mg L^−1^ Cd^2+^) were used in this study. Each treatment included five hydroponic boxes, and each hydroponic box contained six seedlings. An aerobic pump provided continuous aeration during the daytime, and ½ Hoagland solution with Cd treatment and ABA+Cd treatment were renewed every three days. Additionally, 1% ethanol-water (1 mL ethanol was diluted with ultrapure water to the final volume of 100 mL) was used to dissolve ABA, and after this, ultrapure water was added to prepare the ABA stock solution (1 mg mL^−1^); then, 160 μL of ABA stock solution was taken, and ½ Hoagland solution (containing 10 mg L^−1^ Cd^2+^) was added to make the volume up to 800 mL. The Cd^2+^ was supplied as CdCl_2_ into the ½ Hoagland solution, and the seedlings were grown in a greenhouse with a 16 h light/8 h dark cycle at 22 °C. After 10 days of treatment, the seedlings from the hydroponic experiments were harvested for further study.

### 4.3. Measurement of Antioxidative Enzyme Activity

Roots, stems, and leaves were harvested from the Cd−T and ABA+Cd−T samples to measure their SOD and POD activities using a method previously described by Afzal et al. [69]. The tissues collected from four seedlings were mixed into one sample (one biological replicate); each experiment included three biological replicates, and each biological replicate consisted of three technical replicates.

### 4.4. Measurement of MDA Concentration

Roots, stems, and leaves were harvested from the Cd−T and ABA+Cd−T samples to measure their MDA concentration using a method previously described by Chen et al. [70]. The tissues collected from four seedlings were mixed into one sample (one biological replicate); each experiment included three biological replicates, and each biological replicate consisted of three technical replicates.

### 4.5. Measurement of H_2_O_2_ Concentration

Roots, stems, and leaves were harvested from the Cd−T and ABA+Cd−T samples to measure their H_2_O_2_ concentration using a method previously described by Zhang et al. [71]. The tissues collected from four seedlings were mixed into one sample (one biological replicate); each experiment included three biological replicates, and each biological replicate consisted of three technical replicates.

### 4.6. Measurement of Phytohormone Concentration

Roots, stems, and leaves were harvested from the Cd−T and ABA+Cd−T samples to measure their ABA, IAA, and GA_3_ concentrations using a method previously described by You et al. [72]. The tissues collected from four seedlings were mixed into one sample (one biological replicate); each experiment included three biological replicates, and each biological replicate consisted of three technical replicates.

### 4.7. Measurement of Cd^2+^ Concentration

Roots, stems, and leaves were harvested from the Cd−T and ABA+Cd−T samples, soaked in 20 mM EDTA-Na_2_ for 20 min, and washed with deionized water (three times) to remove Cd^2+^ adhered to the surface. The samples were dried at 105 °C for 30 min, followed by drying at 80 °C until the weight was constant. Then, the dry samples were used to measure the Cd^2+^ concentration by ICP-MS, following the method described by Xie et al. [73]. The tissues collected from three seedlings were mixed into one sample (one biological replicate); each experiment included three biological replicates, and each biological replicate consisted of three technical replicates.

### 4.8. Total RNA Extraction, Library Preparation, and De Novo Sequencing

The harvested roots from the Cd−T and ABA+Cd−T samples were frozen in liquid nitrogen for 1 h. Subsequently, the samples were stored at –80 °C to be used in further transcriptome analysis. The tissues collected from three seedlings were mixed into one sample (one biological replicate), and each analysis included three biological replicates. The total RNA was extracted to prepare a library for de novo sequencing using a method previously proposed by Xie et al. [74].

### 4.9. Functional Annotation and Classification

The assembled unigenes were analyzed and annotated against publicly available protein databases, including non-redundant protein sequence database (NR), Swiss-Prot, and eukaryotic orthologous groups (KOG). The BLAST algorithm was used, with an E-value threshold of 1.0 × 10^−5^ [75]. Gene ontology (GO) annotation was performed by Blast2GO software version 2.5 [76], and Kyoto encyclopedia of genes and genomes (KEGG) pathway annotation was performed by the online KEGG Automatic Annotation Server [77].

### 4.10. Analysis and Functional Enrichment of DEGs

The FPKM method was used to determine gene expression. The DESeq (with replicates) estimateSizeFactor function and a negative binomial test were used to identify DEGs between the Cd−T and ABA+Cd−T samples [78]. Significant DEGs were identified using the criteria |Log_2_FoldChange| ≥ 1 and *p*-value ≤ 0.05, followed by the GO function and KEGG pathway enrichment analysis.

### 4.11. Real-Time PCR Analysis of Gene Expression

Roots were harvested from Cd−T and ABA+Cd−T samples to extract RNA for real-time PCR analysis using a previously proposed method [74]. Each reaction was performed in three biological replicates, each with three parallel reactions, using the actin gene of *P. acinosa* as an internal standard. All the primer pairs are listed in Table 3.

### 4.12. Statistical Analysis

All experiments included three biological replicates, and each biological replicate consisted of three technical replicates. The values of each treatment were expressed as means ± SE. The statistical significance of the results was determined using Student’s *t*-test; differences were considered to be significant and highly significant at *p* < 0.05 (*) and *p* < 0.01 (**), respectively. All the statistical analyses were performed using SPSS software version 22.0 (IBM, Chicago, IL, USA), and graphical analysis was performed using Origin Pro 8.0 (OriginLab, Northampton, MA, USA).

## 5. Conclusions

**Figure 11:**
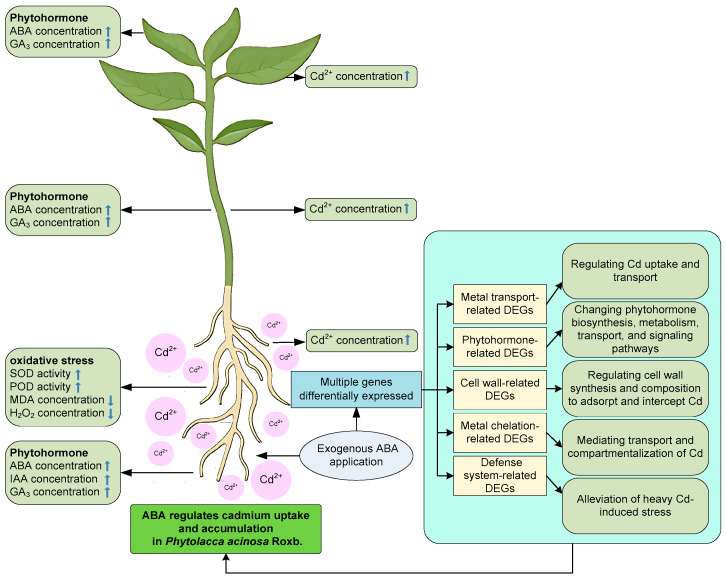
A proposed model for ABA’s regulation of Cd uptake and accumulation in *P. acinosa*. The upward blue arrow represents an increase, and the downward blue arrow represents a decrease.

Overall, this study revealed the molecular mechanisms of ABA-mediated regulation of Cd uptake and accumulation in *P. acinosa*. Physiological and biochemical analysis revealed that exogenous ABA application does not significantly affect plant growth under Cd stress. However, it could alleviate oxidative stress, increase Cd^2+^ concentration, and change the phytohormone concentration in *P. acinosa*. Transcriptome analysis revealed that ABA treatment affected gene expression in *P. acinosa* roots under Cd stress. This study identified 5788 DEGs (2541 up-regulated and 3247 down-regulated). We identified 96 metal transport-related DEGs, 54 phytohormone-related DEGs, 89 cell wall-related DEGs, 113 metal chelation-related DEGs, and 102 defense system-related DEGs that cooperated more closely under exogenous ABA application to regulate Cd uptake and accumulation in *P. acinosa* under Cd stress. A model is proposed in Figure 11.

These results may help to elucidate the mechanisms by which ABA regulates Cd uptake and accumulation in plants, providing novel technical insights and approaches to phytoremediation. The key genes involved in ABA’s regulation of Cd uptake and accumulation in *P. acinosa* must be further analyzed and functionally verified in the future. This may expand our understanding of the molecular regulatory mechanisms involved in heavy metal uptake and accumulation in hyperaccumulators.

## Figures and Tables

**Table 1:** Basic data of transcriptome sequencing.

Sample	Raw Read Number	Raw Base Number	Clean Read Number	Clean Base Number	Valid Bases (%)	Q20 (%)	Q30 (%)	GC (%)
Cd−T1	37,516,010	5,627,401,500	37,184,932	5,549,209,598	98.61%	97.68%	93.16%	46.00%
Cd−T2	43,508,604	6,526,290,600	42,969,078	6,411,399,243	98.23%	97.91%	93.73%	46.00%
Cd−T3	42,372,036	6,355,805,400	41,966,768	6,268,020,644	98.61%	97.84%	93.58%	46.00%
ABA+Cd−T1	37,543,856	5,631,578,400	37,209,394	5,536,724,230	98.31%	97.88%	93.68%	45.00%
ABA+Cd−T2	39,293,478	5,894,021,700	38,817,700	5,787,819,153	98.19%	97.89%	93.70%	47.50%
ABA+Cd−T3	46,979,258	7,046,888,700	46,448,560	6,935,119,640	98.41%	97.89%	93.70%	48.00%

**Table 2:** Expression profiles of ABC transporter families.

Subfamily	ID	Gene	Log_2_FoldChange	Regulation
*ABCA*	DN226612_c1_g3_i5	*ABCA1*	2.64	Up
	DN218393_c2_g1_i5	*ABCA2*	1.97	Up
	DN227994_c8_g1_i3	*ABCA7*	2.34	Up
*ABCB*	DN194339_c0_g1_i1	*ABCB1*	−3.19	Down
	DN228060_c3_g2_i2	*ABCB4*	−2.80	Down
	DN220815_c0_g1_i1	*ABCB8*	4.31	Up
	DN220558_c0_g3_i2	*ABCB15*	2.39	Up
	DN189123_c0_g1_i1	*ABCB17*	4.94	Up
	DN173660_c0_g1_i1	*ABCB19*	4.42	Up
	DN225659_c2_g1_i1	*ABCB20*	−2.13	Down
	DN228060_c3_g1_i9	*ABCB21*	1.49	Up
	DN132622_c0_g1_i1	*ABCB25*	3.76	Up
	DN225703_c4_g1_i1	*ABCB26*	2.93	Up
	DN226871_c9_g1_i19	*ABCB28*	1.87	Up
*ABCC*	DN225934_c6_g2_i1	*ABCC1*	3.45	Up
	DN228942_c1_g1_i6	*ABCC2*	3.12	Up
	DN46026_c0_g1_i1	*ABCC3*	−3.79	Down
	DN222948_c9_g1_i1	*ABCC4*	2.00	Up
	DN228734_c0_g1_i7	*ABCC5*	3.19	Up
	DN218095_c0_g1_i2	*ABCC9*	3.60	Up
	DN226038_c0_g5_i1	*ABCC10*	3.42	Up
	DN224101_c1_g1_i7	*ABCC12*	2.60	Up
	DN200068_c0_g1_i4	*ABCC13*	−3.86	Down
	DN219104_c0_g3_i2	*ABCC14*	3.40	Up
*ABCD*	DN224069_c2_g1_i11	*ABCD1*	2.39	Up
*ABCE*	DN228480_c2_g1_i12	*ABCE2*	2.61	Up
*ABCF*	DN226310_c0_g5_i1	*ABCF1*	2.45	Up
	DN221793_c0_g1_i1	*ABCF3*	3.47	Up
	DN216277_c0_g1_i1	*ABCF4*	2.57	Up
	DN216043_c1_g3_i1	*ABCF5*	1.77	Up
*ABCG*	DN197065_c0_g1_i1	*ABCG2*	2.31	Up
	DN203839_c0_g1_i1	*ABCG3*	2.98	Up
	DN227456_c0_g8_i1	*ABCG4*	3.69	Up
	DN200185_c0_g4_i1	*ABCG6*	2.66	Up
	DN228592_c0_g1_i1	*ABCG7*	1.44	Up
	DN218943_c3_g2_i10	*ABCG11*	−2.34	Down
	DN203802_c0_g1_i1	*ABCG14*	3.95	Up
	DN196886_c0_g1_i2	*ABCG15*	1.91	Up
	DN221450_c0_g1_i2	*ABCG25*	2.26	Up
	DN221568_c5_g1_i7	*ABCG28*	−2.19	Down
	DN226694_c0_g1_i5	*ABCG31*	2.47	Up
	DN228476_c1_g5_i1	*ABCG42*	4.41	Up
	DN224679_c1_g2_i1	*ABCG53*	−1.82	Down
*ABCI*	DN227970_c1_g1_i6	*ABCI1*	2.14	Up
	DN126655_c0_g2_i1	*ABCI6*	3.28	Up
	DN219020_c7_g2_i2	*ABCI17*	2.35	Up
	DN195569_c0_g1_i1	*ABCI19*	−2.28	Down
	DN221951_c1_g1_i2	*ABCI20*	2.78	Up

**Table 3:** The primer pairs for real-time PCR.

ID	Primer Sequences 5′–3′
DN226425_c0_g3_i2	F: CGGGTGGTTGGTTCACAGTA
	R: CCAAGCTAAGTGCCCCATCA
DN220815_c0_g1_i1	F: GTCGTCTTCTCAGGGTGGTG
	R: ATTGGCGTAGGATCGGGTTC
DN221085_c4_g1_i8	F: ATTGCCTCTTGCTCTTCCGT
	R: GCTAGCCTCCTTCATGGGTG
DN192292_c1_g1_i1	F: CACGTGACGTCTTTGGCTAC
	R: AGTATTCCTTCTCGATCCGCC
DN229031_c8_g1_i5	F: TCAACACCGCGCTCATTACT
	R: CCCACCCCTGATAACCAACC
DN175421_c0_g1_i1	F: CTGCAGGTGTCCCGATGATT
	R:CCCATTGCATAGCCACCTCA
DN219478_c1_g1_i4	F: TATCCCCCTGGCGTTTGTTC
	R: GCATTGGGTTGGCGTTCATT
DN220579_c6_g1_i2	F: CCTGCTGATGTTTCCAAGCG
	R: ACTTCTACCCGAGTCCGAGA
DN216324_c5_g3_i1	F: TCGAGAAAGAGGCGAACCTG
	R: GGCGATCAACGGTGGAGATT
DN223975_c4_g1_i1	F: TACTCATGGGGGAGGGGAAG
	R: CCACACTCGATCTGGCATGT
Actin	F: TTGAGCAGGAATCGGAG
	R: TGCTGCTTCCATACCTATC

## Data Availability

The data presented in this study are available from the corresponding author upon reasonable request.
